# COPD in HIV-Infected Patients: CD4 Cell Count Highly Correlated

**DOI:** 10.1371/journal.pone.0169359

**Published:** 2017-01-05

**Authors:** Karine Risso, Francine Guillouet-de-Salvador, Laure Valerio, Pascal Puglièse, Alissa Naqvi, Jacques Durant, Elisa Demonchy, Isabelle Perbost, Eric Cua, Charles-Hugo Marquette, Pierre-Marie Roger

**Affiliations:** 1 Service d’Infectiologie, Centre Hospitalier Universitaire de Nice, Nice, France; 2 Université de Nice-Sophia-Antipolis, Nice, France; 3 Département d’Informations Médicales, Centre Hospitalier de la Dracénie, Draguignan, France; 4 Service de Pneumologie, Centre Hospitalier Universitaire de Nice, Nice, France; Lee Kong Chian School of Medicine, SINGAPORE

## Abstract

**Background:**

COPD is a frequent and significant cause of respiratory morbidity in HIV-infected patients despite the control of HIV. We aimed to analyze the factors correlated with COPD in this population to evaluate the existence of specific indicators of vulnerability in this population.

**Methods and Findings:**

623 HIV-infected outpatients were enrolled during one year. This population was characterised by a dedicated questionnaire and electronic patient records. COPD screening was performed according to recommended spirometric criteria. The prevalence of COPD was 9.0%. Age and smoking were independently correlated with COPD (OR, 1.61 per 10 years increase, *P* = 0.007; OR, 1.28 per 10 pack-year increase, *P* = 0.003, respectively). Body mass index (BMI) and CD4 cell-count were independently and negatively correlated with COPD (OR, 0.78, *P* < 0.001; 0R, 0.77 per 100 cell/mm^3^ increase, *P* < 0.001, respectively). Among COPD patients, 77% did not know their diagnosis. Five COPD-patients never smoked and 44.2% did not have any respiratory symptoms and so were not eligible to perform a spirometry according to the guidelines.

**Conclusions:**

In addition to known risk factors, immune defect through CD4 cell count was independently and strongly correlated with COPD. COPD is largely underdiagnosed and thus unmanaged. However, early management and urgent smoking cessation are essential to improve prognosis. Clinicians’ awareness on the particular vulnerability for COPD in HIV-infected patients is crucial. Moreover, indications to perform conventional spirometry to diagnose COPD may include more parameters than tobacco-smoking and respiratory complaints with a particular concern toward patients with a profound CD4 cell count defect.

## Introduction

COPD will become the third most common cause of death in 2030 in the general population [1]. Early detection and appropriate management is a priority in order to improve patients’ prognosis and quality of life [2].

Human immunodeficiency virus (HIV) infects 150 000 people in France. Eighty-one percent of the patients receive antiretroviral therapy (ART) and HIV viral load is undetectable in 88.5% of cases [3]. In industrialized countries, HIV infection is now considered as a chronic disease in a population with a higher prevalence of various comorbidities [3–5]. International guidelines detailed specific recommendations for cardiovascular, hepatic, metabolic and psychiatric disorders in this population, but did not universally contain specific recommendations on chronic respiratory diseases [3–5].

Yet HIV-infected patients smoke two to three times more than the general population, and have a worrying prevalence of respiratory complaints and lower respiratory tract infections (LRTI) despite effective ART and immune restoration [6–10]. Epidemiological studies in the ART era showed more COPD among HIV-infected people [11–15]. In addition to known risk factors for COPD (tobacco smoking, age and body mass index), involvement of HIV-specific risk factors remains suspected. To date, studies assessing specific associations between HIV markers and HIV related parameters with COPD have yield contradictory results [12,13,16–20]. In order to explore this association, we studied, in a large cohort of HIV-infected outpatients, the prevalence of COPD and the related factors including all the factors previously tested in the literature between HIV and COPD.

## Materials and Methods

### Design and study population

This prospective monocentric cross sectional study took place in the Infectious Diseases Department of the Nice University Hospital where a cohort of 2453 HIV-infected patients is followed up with 93% of patients under ARV therapy and 80% of patients with a undetectable viral load. All the adult patients consulting at the outpatient clinic during 3 randomly selected days per week, from January 1^st^, to December 31^st^ 2012 were eligible. Patients with recent LRTI (≤ 2 months), or with mental or physical incapacity to perform pulmonary function test (PFT) were excluded.

### Screening and data collection

Patients first completed a dedicated questionnaire (S1 Appendix) with the assistance of a medical student or a nurse and performed a rapid PFT with a hand-held COPD-6 spirometer. The questionnaire searched for respiratory symptoms (chronic bronchitis, recurrent acute bronchitis, dyspnea), history of hospitalization for respiratory-related conditions, known COPD or chronic bronchopathy, smoking history, use of illicit drugs (cannabis, intra-venous drug use), occupational respiratory exposure and socio-economical status. To characterize COPD, according to recent definitions, COPD frequent exacerbator phenotype was defined as a patient with 2 or more acute bronchitis per year [1,21]. Patient-orientated definitions of chronic bronchitis, recurrent acute bronchitis and dyspnea used in our questionnaire had previously been tested for their understandability and validated as conform by pneumologists [1,2,21].

All patients with respiratory symptoms, a previous mentioned COPD or chronic bronchopathy diagnosis, a history of hospitalization for a respiratory-related condition, or an abnormal COPD-6 test, underwent a conventional spirometry performed by a pneumologist (FGdS and KR).

Data concerning HIV infection (date of HIV infection, AIDS-defining diagnosis according to Centers for Disease Control and Prevention staging, nadir CD4, CD4 and CD8 cell count within the last 6 months with CD4/CD8 ratio and HIV RNA load, treatment history, previous respiratory opportunistic infections) and comorbidities were collected from the Nadis^®^ electronic patient medical record [22].

### Definition of COPD

PFT tests were performed following the American Thoracic Society (ATS) / European Respiratory Society (ERS) guidelines [23,24]. According to the GOLD guidelines, diagnosis of COPD was defined as a Forced Expiratory Volume in one second (FEV1) / Forced Vital Capacity (FVC) < 70% after bronchodilators test [1].

### Ethics

The Nice University Hospital Ethics Committee Board review approved the study. All patients provided with their written informed consent.

### Statistical analysis

Based on an obstructive lung disease (OLD) prevalence rate of 7.5% in the French general population, a precision of 2% and a level of significance of 5%, the sample size required to estimate the prevalence of COPD in our population was 666 patients. Continuous variables are presented as mean ± standard deviation (SD). Student’s t-test was used to compare continuous variables and Chi-squared and Fisher’s exact tests were used for discrete variables. Statistical significance was considered at *P* < 0.05. We first estimated the prevalence of COPD and its 95% confidence interval (CI). Then, to identify factors independently correlated with COPD, we compared COPD patients to non-COPD patients using a logistic regression (LR) model introducing the usual risk factors for COPD (age per ten years increase, smoking per 10 pack years increase, body mass index), drug exposure (ever cannabis user and ever intravenous drug user), and several parameters of HIV infection (HIV infection duration, CD4 cell count, Nadir CD4 cell count, HIV RNA viral load, duration of HAART exposure for nucleoside reverse transcriptase inhibitor, non-nucleoside reverse-transcriptase inhibitors and protease inhibitor, HBV or HCV co-infection, mycobacterial lung infection). The statistical analysis was performed using SPSS© 14.0 software.

## Results

### Study population characteristics

**Fig 1:**
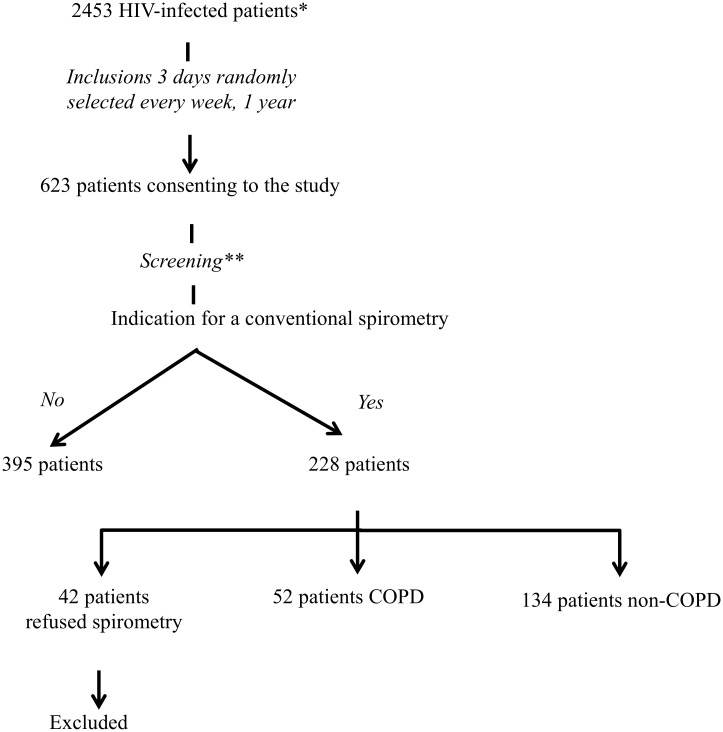
Study’s flowchart. * Cohort of HIV-infected patients consulting in infectious department during 1 year. ** Dedicated questionnaire + Hand-Held spirometer.

The study’s flowchart is presented in Fig 1. We enrolled 623 HIV-infected patients. These patients did not differ from our entire cohort (n = 2453) for age, gender distribution (data not shown), percentage of patients under-ARV therapy (93.5%), with undetectable viral load (80%) or CD4> 500 (64%). Forty-two patients screened by questionnaire or COPD-6 with an indication to perform a conventional spirometry refused to undergo the exam and were therefore excluded. Characteristics of patients included and excluded in our study are detailed in Table 1. Briefly, 73.8% of patients were men, mean age was 48 years with a mean duration of HIV infection of 15.5 years. At the time of the study, 93.5% of patients were receiving ART, with an undetectable HIV viral load for 85.2% and a mean CD4 cell-count of 622 cells/mm^3^. Twenty percent of patients were past smokers, 51% were current smokers. In our institution, all the patients with CD4 cell count < 200/mm3 or < 15% were receiving pneumocystis pneumonia prophylaxis. Details concerning previous respiratory opportunistic infections, comorbidities, socio-economic status and educational level of patients are to be found in supporting information (S1 Table).

**Table 1:** Baseline characteristics of study cohort: comparison included/excluded.

Parameters	Patients included n = 581	Patients excluded n = 42	*P* value
**Demographic characteristic**			
Age (years)	48.3 ± 9.9	44.4 ± 10.2	**0.013**
Male Gender	429 (73.8%)	26 (61.9%)	0.092
**Clinical characteristics**			
Respiratory symptoms	136 (23.6%)	19 (45.2%)	**0.005**
Chronic bronchitis symptoms	45 (7.7%)	5(11.9%)	0.563
Recurrent acute bronchitis	50 (8.6%)	8 (19%)	**0.045**
Dyspnea	100 (17.2%)	13 (31%)	0.068
Hosp. for respiratory condition	32 (5.5%)	7 (16.7%)	**0.012**
Previous LRTI	205 (35.3%)	19 (45.2%)	0.194
Previous CABP	45 (9.8%)	6 (14.3%)	0.42
Known as chronic bronchitis	27 (4.7%)	5 (11.9%)	0.057
BMI (Kg/m^2^)	23.5 ± 3.6	23.2 ± 4.3	0.69
**Toxic exposure**			
Current smoker	295 (50.8%)	25 (59.5%)	0.273
Current or past smoker	417 (71.8%)	34 (81.0%)	0.199
Pack-years history	16.1 ± 17.9	21. 4± 25.6	0.26
Current/past cannabis user	226 (40.4%)	18 (45.0%)	0.564
IDU	103 (18.3%)	6 (15.0%)	0.601
Professional resp. exposure	141 (24.7%)	13 (31.7%)	0.317
**HIV disease**			
HIV infection duration (years)	15.5 ± 8.6	14.9 ± 9.3	0.681
CDC stage			0.605
A	350 (60.2%)	22 (52.4%)	
B	93 (16.0%)	8 (19.0%)	
C	138 (23.8%)	12 (28.6%)	
CD4 T-cell count (cells/mm^3^)	622 (291)	584 (412)	0.562
<200 cells/mm^3^	32 (5.5%)	8 (19.0%)	**0.003**
200–349 cells/mm^3^	59 (10.2%)	5 (11.9%)	
350–499 cells/mm^3^	111 (19.1%)	10 (23.8%)	
>500 cells/mm^3^	379 (65.2%)	19 (45.2%)	
CD4/CD8 cell ratio	0.79 ± 0.51	0.64 ± 0.42	0.069
HIV RNA (log_10_ cp/ml)	1.85 ± 0.77	2.22 ± 1.28	0.075
Undetectable HIV RNA	494 (85.2%)	30 (71.4%)	**0.018**
Nadir CD4 cell count (cells/mm^3^)	255 ± 189	194 ± 162	**0.043**
HBV and/or HCV infection	193 (33.2%)	11 (26.2%)	0.349
**HAART exposure**			
Current HAART	543 (93.5%)	38 (90.5%)	0.517
HAART naïve	24 (4.1%)	2 (4.8%)	0.692
NRTI duration (month)	117.4 ± 81.2	107.1 ± 70.5	0.395
NNRTI duration (month)	37.0 ± 49.5	32.3 ± 48.2	0.54
PI duration (month)	63.5 ± 63.9	55.5 ± 54.5	0.415

Data are expressed as % (No./total No.) or mean ± standard deviation.

Abbreviations: Hosp. hospitalization, LRTI lower respiratory tract infection, CABP community-acquired bacterial pneumonia, BMI body mass index, IDU intravenous drug use, resp. respiratory, CDC Centers for Disease Control and Prevention, Undetectable HIV RNA < 40 cp/ml, HAART highly active antiretroviral therapy, NRTI nucleoside reverse transcriptase inhibitor, NNRTI non-nucleoside reverse-transcriptase inhibitors, PI protease inhibitor.

### COPD among HIV-infected patients

The prevalence of COPD was 9% (52/581) (95% CI, 6.6%—11.3%). One hundred eighty-four conventional spirometries were performed after screening, enabling the diagnosis of 64 OLD (35%) and 52 COPD (28%). Previous hospitalization for a respiratory-related condition was more frequent among COPD-patients compared to non-COPD patients (63.5% vs. 32.5%, *P* < 0.001) Table 2. Previous community-acquired bacterial pneumonia (CABP) was also more frequent among COPD-patients (23.1% vs. 8.5%, *P* = 0.001).

**Table 2:** Comparison COPD-patients and non-COPD patients.

Parameters	Univariate analysis			Multivariate analysis	
	Non-COPD	COPD	*P* Value	OR^d^ (95% CI)	*P* Value
	(529)	(52)			
**Demographic characteristics**					
Age (years) ^#^	47.9 ± 9.8	52.5 ± 9.7	**0.001**	**1.61**^a^ **(1.14–2.28)**	**0.007**
Male Gender	389 (73.5%)	40 (76.9%)	0.596		
BMI (Kg/m^2^) ^#^	23.7 ±3.6	21.5 ± 3.4	**<0.001**	**0.78 (0.70–0.89)**	**<0.001**
**Toxic exposure**					
Current smoker	261 (49.3%)	34 (65.4%)	**0.027**		
Current or past Smoker	370 (69.9%)	47 (90.4%)	**0.002**		
Pack-year history^#^	15.4 ± 17.5	23.6 ± 19.4	**0.001**	**1.28**^b^ **(1.09–1.50)**	**0.003**
Current/past Cannabis use^#^	199 (38.9%)	27 (55.1%)	**0.028**		
IDU^#^	85 (16.6%)	18 (35.3%)	**0.001**		
Professional resp. exposure	128 (24.7%)	13 (25.0%)	0.957		
**Clinical characteristics**					
Respiratory symptoms	107 (20.3%)	29 (55.8%)	**<0.001**		
Chronic bronchitis symptoms	31 (6%)	14 (27%)	**<0.001**		
Recurrent acute bronchitis	37 (7%)	12 (23.1%)	**<0.001**		
Dyspnea	78 (14.7%)	23 (44.2%)	**<0.001**		
Hosp. for respiratory condition	19 (3.6%)	13 (25.0%)	**<0.001**		
Previous LRTI	172 (32.5%)	33 (63.5%)	**<0.001**		
Previous CABP	45 (8.5%)	12 (23.1%)	**0.001**		
HBV and/or HCV infection^#^	167 (31.6%)	26 (50.0%)	**0.007**		
Depression	103 (19.5%)	19 (36.5%)	**0.004**		
**HIV story**					
HIV infection duration (years) ^#^	15.2 ± 8.5	18.7 ± 8.5	**0.005**		
CDC stage			0.376		
A	320 (60.5%)	30 (57.7%)			
B	87 (16.4%)	6 (11.5%)			
C	122 (23.1%)	16 (30.8%)			
CD4 cell count (cells/mm^3^) ^#^	634 ± 294	497 ± 232	**0.001**	**0.77**^c^ **(0.68–0.88)**	**<0.001**
<200 cells/mm^3^	28 (5.3%)	4 (7.7%)	**0.008**		
CD4/CD8 cell ratio	0.79 ± 0.51	0.71 ± 0.44	0.271		
CD8 cell count (cells/mm^3^)	939 ± 467	830 ± 548	0.10		
HIV RNA (log_10_ cp/ml) ^#^	1.87 ± 0.79	1.71 ± 0.54	**0.054**	0.59 (0.32–1.08)	0.088
Undetectable HIV RNA	446 (84.5%)	48 (92.3%)	0.129		
Nadir CD4 cell count (cells/mm^3^) ^#^	262 ± 191	188 ± 155	**0.007**		
**HAART exposure**					
HAART naïve	24 (4.5%)	0 (0.0%)	0.154		
NRTI (months) ^#^	116.3 ± 81.5	128.5±77.9	0.465		
NNRTI (months) ^#^	35.7 ± 48.1	50.7 ± 60.8	0.114		
PI (months)^#^	63.2 ± 63.8	66.9 ± 65.1	0.863		

Data are expressed as % (No./total No.) or mean ± standard deviation,

^#^ parameters included in the multivariate regression analysis,

^a^ per 10 year-increase in age,

^b^ per 10 pack-year-increase,

^c^ per 100 cells/ mm^3^ increase,

^d^ only significant results are shown.

Abbreviations: COPD chronic obstructive pulmonary disease, OR odds ratio, CI confidence interval, BMI body mass index, IDU intravenous drug use, Hosp. hospitalization, LRTI lower respiratory tract infection, CABP community-acquired bacterial pneumonia, CDC centers for disease control and prevention, undetectable HIV RNA < 40 cp/ml, HAART highly active antiretroviral therapy, NRTI nucleoside reverse transcriptase inhibitor, NNRTI non-nucleoside reverse-transcriptase inhibitors, PI protease inhibitor.

### COPD diagnosis and management

Screening tests results are summarized in Supporting Information (S2 Table). Among the 52 COPD-patients, 40 (77%) did not know they had a bronchopathy and 47 (90%) had never performed a spirometry. Only five patients (9.6%) were treated for COPD.

### Factors associated with COPD in HIV-infected patients

In the univariate analysis (Table 2), COPD-patients were significantly older (52.5 years vs. 47.9 years; *P* = 0.001), more inactive, presenting long-term illness or disability (*P* = 0.003) (S1 Table). They had a significantly lower BMI (20.7 kg/m^2^ vs. 23.4 Kg/m^2^, *P* < 0.001). COPD-patients were more frequently current or past smokers (90.4% vs. 69.9%) with higher smoking exposure in pack-year (23.6 vs. 15.4). Exposure to cannabis and IDU was significantly more common among COPD-patients (55.1% vs. 38.9%, *P* = 0.028 and 35.3% vs. 16.6%, *P* = 0.001, respectively). Regarding HIV parameters, COPD-patients had a significantly lower CD4 cell-count (497 vs. 634 cells/mm^3^, *P =* 0.001) and lower mean nadir CD4 cell-count (188 cells/mm^3^ vs. 262 cells/mm^3^; *P =* 0.007). Among associated comorbidities, we found in COPD-patients higher frequencies of co-infection (active or previous) with hepatitis B or C viruses (50.0% vs. 31.6%, *P =* 0.007) and depression (36.5% vs. 19, 5%, *P =* 0.004) (Table 2).

According to multivariate analysis, age (OR, 1.61 per 10 years increase, *P = 0*.*007*), BMI (OR, 0.78, *P < 0*.*001*), smoking (OR, 1.28 per 10 pack-years increase, *P = 0*.*003*) and CD4 cell-count (OR, 0.77 per 100 CD4 cell/mm^3^ increase, *P < 0*.*001*) remained significantly and independently associated with COPD (Table 2).

## Discussion

Our 623 patients randomly included were representative for age, sex ratio and HIV infection control of our entire cohort of 2453 patients. They were also representative of the HIV-infected patients population currently followed up in most industrialized countries for age and sex ratio [3,25]. This study showed a prevalence of 9.0% (95% CI, 6.6%—11.3%) for COPD and identified a CD4 cell count to be strongly and independently correlated with COPD.

The prevalence of COPD worldwide varies significantly across countries [1,2,26]. In France, the prevalence of obstructive lung disease in the general population (including asthma and COPD) was estimated at 7.5% [27]. This prevalence, lower than the one found in our study, was evaluated in a much older population (60±10 years) than ours [27].

A 2006 study by Crothers *et al*. alerted clinicians to an increased prevalence of COPD in HIV-infected patients on the basis of self-reported diagnosis (15% vs. 12%, *P = 0*.*04)* [11]. Since the publication of this result, 5 high quality studies using spirometric criteria have found COPD rates between 6% and 21% in groups of 65 to 400 HIV outpatients representative of patients commonly treated in consultation units in the United-States, Spain, Italy and Nigeria [12,13,16,19,20]. Differences in prevalence were expected due to dissimilarities of methodology, age, smoking, histories and country-related respiratory exposure usually observed between the countries, but all of these studies confirmed similar high frequency of COPD. COPD induced a worrying respiratory morbidity as attested by more frequent hospitalization for a respiratory condition (25% *vs*. 3.6%, P < 0.001) and previous lower respiratory tract infection (63.5% *vs*. 32.5% P < 0.001) (Table 2).

Aging and smoking were independently correlated with COPD (Table 2). These are the two most frequent risk factors for COPD in the general population, with cigarette smoking accounting for 95% of toxic respiratory exposure responsible for COPD in industrialized countries [1]. We confirmed this known high exposure to tobacco smoking in HIV-infected patients (50.8% of current smokers in our study, compared to 33% in the general French general population) [6,7,28]. These results are alarming, especially because the attributable risk of death associated with smoking among HIV-infected patients is doubled compared to uninfected population [29].

Regarding other factors correlated with COPD (Table 2), low BMI, which is known to be correlated with a poor COPD prognosis, was also correlated with COPD as in the general population [30]. We did not find any independent correlation with the use of cannabis, IDU, depression, inactive status or long-term illness, probably because these factors were associated with smoking [31]. We did not find any correlation between ART exposure and COPD, nor between hepatitis and COPD, contrary to some previous studies using smaller populations [10,12,14,32]. Some previous studies also discussed an association between pneumocystis pneumonia or tuberculosis pneumonia with COPD [10, 19]. Our study did not find any association with these pathologies, but their low frequency in our population did not allow for any meaningful conclusions on this point (S1 Table).

More age-associated diseases characterize HIV-infected patients. Immune defects including nadir CD4, CD4 cell count, and low CD4/CD8 ratio are involved in a chronic inflammatory state. This contributes to accelerated aging and the development of cardiovascular, rheumatological, renal, neurological comorbidities [33,34,35]. We identified 2 markers of HIV infection negatively correlated with COPD (Table 2): nadir CD4 cell-count (188 CD4 cells/mm^3^ in COPD-patients *vs* 262 CD4 cells/mm^3^ in non COPD-patients, *P* = 0.007) in the univariate analysis only, and most recent CD4 cell-count in both the univariate and multivariate analyses (OR, 0.77 for each 100 cells/mm^3^ increase; 95% CI, 0.68–0.88). *Madeddu et al*. concluded that HIV was probably a risk factor of COPD independently of smoking and age [12]. Ours results suggest that HIV could increase the risk of COPD through CD4 cell count depletion. The implication of CD4 T-cell count in the pathogenesis of OLD as been suggested in the literature [10,11,36,37]. In two recent studies, Drummond and Shirley have shown an accelerated decline of respiratory function (forced expiratory volume in one second (FEV1) and forced vital capacity (FVC)) in HIV-infected people with lower CD4 cell counts [18,20]. In his cohort of 303 HIV-infected patients, Drummond observed, contrary to our results, that this association with CD4 cell count disappeared after adjustment for HIV viral load suggesting that HIV viral load was more determinant in respiratory decline than CD4 cell level [18]. These results again confirm a plausible role played by poor control of HIV disease in COPD pathogenesis. However, the study was probably underpowered for testing the specific role of CD4 cell count because the population was limited to intravenous drug users, heavy tobacco smoker and various drug users with very poor control of their HIV-disease, including a global low level of CD4 cells (323/mm3 *vs*. 622 CD4 cells/mm^3^ in our population) and a frequent high HIV viral load [17,18]. Studying CD4 cells in another body fluid compartment, *L*. *Popescu et al*. recently observed a significant correlation between CD4 cell count defect in bronchiolo-alveolar lavage liquid and COPD in HIV-infected patients [38]. Our study confirmed literature suspicion and found for the first time in a large cohort of patient a significant and independent correlation between COPD and CD4 cell count defect. This link between CD4 cell count and COPD is also supported by various pathogenetic explanations in the literature. A CD4 cell defect could favor COPD through bronchial colonization especially by *pneumocystis jiroveci* and secondary bronchial inflammation, dysimmune processes and accelerated aging [39–42]. Our study did not explore associated functional modifications in CD4 cells, but other interesting studies have suggested that tobacco smoking could impair T-cells function in HIV-infected patients [43,44].

Moreover, COPD is frequently not screened, and is under-diagnosed and ill-managed as a consequence. Seventy-seven percent of our COPD-patients were unaware of having a bronchopathy and 90% had never been previously tested or treated. Diagnosis of COPD reinforces the recommendation to quit smoking and enables clinicians to prescribe an appropriate inhaled treatment. Interactions between inhaled corticosteroid and protease inhibitors (PI) is no longer a problem because their indication is now limited to a small subset of COPD patients (COPD GOLD 3 or 4 with frequent exacerbations) and several alternative treatments to PI are available if inhaled corticosteroids are required [1, 45]. Diagnosis of COPD also implicates a specific survey of respiratory function and lung cancer screening [1, 46].

International guidelines advocate COPD-screening for any patient older than 35 years with respiratory complaints (exertional breathlessness, chronic cough, regular sputum production, frequent winter ‘bronchitis’ or wheezing) and a history of exposure to disease risk factors (e.g., current or former smoker) [1,47]. Under-diagnosis of COPD in the general population is a well-known problem and has also been identified in HIV-infected patient populations [1,13,16]. As patients are frequently asymptomatic (44.2% of our COPD patients), the guidelines and criteria for COPD screening appear to be insufficient. The existence of an independent correlation between CD4 cell count and COPD should suggest to clinicians to be aware of COPD risk for patients with less than 200 CD4 cells/mm^3^ and particularly if these patients are smokers. Further studies are required to identify more appropriate strategies to screen for COPD in this population.

Our study has some limitations: we may have underestimated the prevalence of COPD since the 42 patients screened by questionnaire or COPD-6 who refused to undergo conventional spirometry present a high risk of COPD according to subsequent results (see Tables 1 and 2). Moreover, exclusion of all patients with a recent history of LRTI (which might interfere with spirometric results) might have led to underestimating the prevalence of COPD. Therefore, for organizational and budget reasons, we were not able to perform a conventional spirometry for all patients and we applied a preliminary screening using a questionnaire and a hand-held spirometer. These tests were successful in detecting high-risk populations (S2 Table) but are known to have an insufficient sensibility and have probably under-estimate the real prevalence of COPD in our population [48]. We also did not collect the reasons why some patients refused to participate in our study, and we have no data on smoking exposure for our entire cohort, which could have provided a useful comparison.

In conclusion, this study shows that in HIV-infected people, smoking exposure, aging and CD4 cell count is associated with COPD. Moreover, we suggest that diagnosis of this disease, which is frequently asymptomatic at its beginning, could be improved by integrating vulnerability to COPD in connection to CD4 cell count defect into strategies for COPD-screening.

## Supporting Information

S1 Appendix COPD study’s questionnaire (translated from French language). (DOCX)

S1 Table Comparison COPD / non-COPD patients for all parameters analyzed during study. Data are expressed as % (No./total No.) or mean ± standard deviation, # parameters included in the multivariate regression analysis, a per 10 year-increase in age, b per 10 pack-year-increase, c per 100 cells/ mm3 increase.
Abbreviations: COPD chronic obstructive pulmonary disease, OR odds ratio, CI confidence interval, BMI body mass index, IDU intravenous drug use, Hosp. hospitalization, LRTI lower respiratory tract infection, CABP community-acquired bacterial pneumonia, CDC centers for disease control and prevention, undetectable HIV RNA < 40 cp/ml, HAART highly active antiretroviral therapy, NRTI nucleoside reverse transcriptase inhibitor, NNRTI non-nucleoside reverse-transcriptase inhibitors, PI protease inhibitor, pn. Pneumonia, mycobac mycobacteria.
(DOCX)

S2 Table Results of screening test for the 184 conventional pulmonary function testing performed. (DOCX)
