# Supplementary material for: COPD in HIV-Infected Patients: CD4 Cell Count Highly Correlated
Source: PLoS One. 2017 Jan 5;12(1):e0169359. doi: 10.1371/journal.pone.0169359 (PMC5215875; doi:10.1371/journal.pone.0169359)
Supplement: S1 Appendix — (DOCX) [file pone.0169359.s001.docx]

**S1 Appendix. COPD study’s questionnaire (translated from French language)**

Read carefully questions concerning your respiratory symptoms and your risk factor of respiratory disease and try to answer precisely.

**Weight, size:** Your weight _ _ _ kilograms Your size _ _ _meter

**1- If we ask you “do you spit?” you will answer (only one ANSWER)**

Yes I spit at last one time a day, at least 3 months per year, since 2 years

No I don’t spit, or very occasionally

**2- If we ask you if you are breathless, you will answer (only one ANSWER)**

Yes I’m sometimes breathless if I climb 2 floors up the stairs

Yes I’m currently breathless if I walk fast or if I climb

Yes I’m currently breathless if I walk on a flat area at the same speed as somebody as old as me

Yes When I walk, I usually have to stop to catch my breath after several minutes or after 100 meters

Yes I’m breathless even for a lesser effort

No No I am not concerned with the situations mentioned above, breathlessness is not a problem for me.

**3- Were you prescribed antibiotics or corticoids** (solupred®, cortancyl®, prednisolone®, celestene®) **for a bronchitis, and that at least twice during the previous year?** Yes No

**4- Have you already been hospitalized for a respiratory condition?** YES NO

**5 – Have some doctors already said to you that you have a chronic bronchitis (or smoker’s chronic bronchitis or, COPD)?**

YES NO

**If yes**: Have you already made an exam to test your breath (spirometry)? Yes No

Do you take a treatment for your Breath/ for your respiration*? Yes No

* exemple of treatment for respiratory disease: Sérétide®, Symbicort®, Ventoline®, Bécotide®, Béclojet®, Prolair®, Qvar®, Béclométasone®, Béclone®, Pulmicort®, Flixotide®, Miflonil®, Bronchodual®, Combivent®, Atrovent, , Spiriva, Asmabec®, Bemedrex®, Miflasone®, Onbrez®, A Spir®, Nexxair®, Ecobec®, Timos®, Formoair®, Breezhaler®, Atimos®, Formoair®, Tersigat®, Dilatrane®, Euphylline®, Theophylline®, Tédralan®, Xanthium®, Trentadil®, Singulair®

**Smoking**

Do you smoke at the moment? Yes No

If you smoke now or were a smoker in the past:

- At what age did you begin? ………. years

- How many packs of cigarettes do you smoke or did you smoke per day on average? **(only one answer)**

- less than half a pack per day

- between half and one pack per day

- between one and two packs per day

- more than 2 packs a day

If you have stopped smoking: at what age did you give up ? …….years old

**Cannabis**

Do you actually smoke regularly cannabis (at least one joint a week) Yes No

If yes, on average: - between a joint a week and 1 joint a day

- between 1 and 5 joints a day

- more than 5 joints day

In the past have you regularly (at least one joint a week) during a period, smoke cannabis? Yes No

Do you actually or do you have in the past, regularly (at least one time a week) use intravenous drug injection? Yes No

**Professional exposure:**

Have you been exposed in the past for professional reasons to dust, fumes, (agriculture, mine, textile industry…)

Or are you being exposed now? Yes No

**Professional activity**

- Working person - Active but on maternity leave

- Working but actually on sick leave - Unemployed

- Student - Military

- Pensioner

- Disabled

**School level**

- No diploma - Primary school - Secondary school

- High school - College/university

**COPD-6 test**

FEV1 (ml) : _ _ _ FEV6 (ml) : _ _ _ FEV1/FEV6 : _ _

COPD probability:

- Low - Moderate - High

**Incident :** Yes No

**If yes: did the test need to be stopped ?:** Yes No

***Indication to perform a Spirometry if one of the following conditions:***

*- Patient answers “yes” to one of the first five questions*

*- Probability “moderate” or “high” of COPD on COPD-6 test*
