# Supplementary material for: COPD in HIV-Infected Patients: CD4 Cell Count Highly Correlated
Source: PLoS One. 2017 Jan 5;12(1):e0169359. doi: 10.1371/journal.pone.0169359 (PMC5215875; doi:10.1371/journal.pone.0169359)
Supplement: S1 Table — Data are expressed as % (No./total No.) or mean ± standard deviation, # parameters included in the multivariate regression analysis, a per 10 year-increase in age, b per 10 pack-year-increase, c per 100 cells/ mm3 increase. Abbreviations: COPD chronic obstructive pulmonary disease, OR odds ratio, CI confidence interval, BMI body mass index, IDU intravenous drug use, Hosp. hospitalization, LRTI lower respiratory tract infection, CABP community-acquired bacterial pneumonia, CDC centers for disease control and prevention, undetectable HIV RNA < 40 cp/ml, HAART highly active antiretroviral therapy, NRTI nucleoside reverse transcriptase inhibitor, NNRTI non-nucleoside reverse-transcriptase inhibitors, PI protease inhibitor, pn. Pneumonia, mycobac mycobacteria. (DOCX) [file pone.0169359.s002.docx]

**S1 Table. Comparison COPD / non-COPD patients for all parameter analyzed during study**

|  | **Univariate analysis** | | | **Multivariate analysis** | |  |
| --- | --- | --- | --- | --- | --- | --- |
| **Parameter** | **non - COPD (529)** | **COPD (52)** | **P Value** | **OR (95% CI)** | **P Value** | |
| **Demographics characteristics** | | |  |  |  | |
| Age (years) # | 47.9 ± 9.8 | 52.5 ± 9.7 | **0.001** | **1.61a (1.14-2.28)** | **0.007** | |
| Male Gender | 389 (73.5%) | 40 (76.9%) | 0.596 |  |  | |
| BMI (Kg/m2) # | 23.7 ±3.6 | 21.5 ± 3.4 | **<0.001** | **0.78 (0.70-0.89)** | **<0.001** | |
| Professional category | |  | **0.003** |  |  | |
| Working | 285 (53.9%) | 16 (30.8%) |  |  |  | |
| Long-term illness or invalidity | 114 (21.6%) | 20 (38.5%) |  |  |  | |
| No-working | 130 (24.6%) | 16 (30.8%) |  |  |  | |
| School level |  |  | 0.313 |  |  | |
| Without diploma | 75 (14.2%) | 4 (7.7%) |  |  |  | |
| Primary school | 47 (8.9%) | 3 (5.8%) |  |  |  | |
| Secondary education | 160 (30.2%) | 22 (42.3%) |  |  |  | |
| High school certificate | 116 (21.9%) | 9 (17.3%) |  |  |  | |
| University | 131 (24.8%) | 14 (26.9%) |  |  |  | |
| **Toxic exposure** | |  |  |  |  | |
| Current smoker | 261 (49.3%) | 34 (65.4%) | **0.027** |  |  | |
| Current or past Smoker | 370 (69.9%) | 47 (90.4%) | **0.002** |  |  | |
| Smoking duration (years) | 18.3 ± 1.5 | 29.8 ± 1.3 | **<0.001** |  |  | |
| Pack-years history # | 15.4 ± 17.5 | 23.6 ± 19.4 | **0.001** | **1.28b (1.09-1.50)** | **0.003** | |
| Current cannabis use | 65 (12.3%) | 13 (25.5%) | **0.009** |  |  | |
| Current or past Cannabis use# | 199 (38.9%) | 27 (55.1%) | **0.028** |  |  | |
| IDU# | 85 (16.6%) | 18 (35.3%) | **0.001** |  |  | |
| Professional resp. exposure | 128 (24.7%) | 13 (25.0%) | 0.957 |  |  | |
| **Clinical characteristics** | |  |  |  |  | |
| Respiratory symptoms | 107 (20.3%) | 29 (55.8%) | **<0.001** |  |  | |
| Chronic bronchitis symptoms | 31 (6%) | 14 (27%) | **<0.001** |  |  | |
| Recurrent acute bronchitis | 37 (7%) | 12 (23.1%) | **<0.001** |  |  | |
| Dyspnea | 77 (14.7%) | 23 (44.2%) | **<0.001** |  |  | |
| MMRC scale 0 | 42 (54.5%) | 8 (34.8%) |  |  |  | |
| MMRC scale 1 | 27 (35.1%) | 10 (43.5%) |  |  |  | |
| MMRC scale 2 | 0 | 0 |  |  |  | |
| MMRC scale 3 | 2 (2.6%) | 0 (0.0%) |  |  |  | |
| MMRC scale 4 | 6 (7.8%) | 5 (21.7%) |  |  |  | |
| Hosp. for respiratory condition | 19 (3.6%) | 13 (25.0%) | **<0.001** |  |  | |
| Previous LRTI | 172 (32.5%) | 33 (63.5%) | **<0.001** |  |  | |
| Previous CABP | 45 (8.5%) | 12 (23.1%) | **0.001** |  |  | |
| **HIV story** |  |  |  |  |  | |
| HIV infection duration (years) # | 15.2 ± 8.5 | 18.7 ± 8.5 | **0.005** |  |  | |
| CDC stage |  |  | 0.376 |  |  | |
| A | 320 (60.5%) | 30 (57.7%) |  |  |  | |
| B | 87 (16.4%) | 6 (11.5%) |  |  |  | |
| C | 122 (23.1%) | 16 (30.8%) |  |  |  | |
| CD4 T-cell count (cells/mm3) # | 634 ± 294 | 497 ± 232 | **0.001** | **0.77c (0.68-0.88)** | **<0.001** | |
| <200 cells/mm3 | 28 (5.3%) | 4 (7.7%) | **0.008** |  |  | |
| 200-349 cells/mm3 | 47 (8.9%) | 12 (23.1%) |  |  |  | |
| 350-499 cells/mm3 | 101 (19.1%) | 10 (19.2%) |  |  |  | |
| >500 cells/mm3 | 353 (66.7%) | 26 (50.0%) |  |  |  | |
| CD4/CD8 T-cell ratio | 0.79 ± 0.51 | 0.71 ± 0.44 | 0.271 |  |  | |
| CD8 T-cell count (cells/mm3) | 939 ± 467 | 830 ± 548 | 0.10 |  |  | |
| HIV RNA (log10 cp/ml) # | 1.87 ± 0.79 | 1.71 ± 0.54 | **0.054** | 0.59 (0.32-1.08) | 0.088 | |
| Undetectable HIV RNA | 446 (84.5%) | 48 (92.3%) | 0.129 |  |  | |
| NADIR CD4 (cells/mm3) # | 262 ± 191 | 188 ± 155 | **0.007** |  |  | |
| HAART exposure | |  |  |  |  | |
| HAART naïve | 24 (4.5%) | 0 (0.0%) | 0.154 |  |  | |
| NRTI duration (months) # | 116.3 ± 81.5 | 128.5 ± 77.9 | 0.465 |  |  | |
| NNRTI duration (months) # | 35.7 ± 48.1 | 50.7 ± 60.8 | 0.114 |  |  | |
| PI duration (months) # | 63.2 ± 63.8 | 66.9 ± 65.1 | 0.863 |  |  | |
| **Comorbidities** | |  |  |  |  | |
| HBV and/or HCV infection# | 167 (31.6%) | 26 (50.0%) | **0.007** |  |  | |
| Hypertension | 82 (15.5%) | 9 (17.3%) | 0.732 |  |  | |
| Asthma | 13 (2.5%) | 3 (5.8%) | 0.165 |  |  | |
| Renal failure | 16 (3.0%) | 4 (7.7%) | 0.094 |  |  | |
| Dyslipidemia | 180 (34.0%) | 20 (38.5%) | 0.521 |  |  | |
| Stroke | 5 (0.9%) | 1 (1.9%) | 0.432 |  |  | |
| Depression | 103 (19.5%) | 19 (36.5%) | **0.004** |  |  | |
| Cancer | 53 (10.0%) | 8 (15.4%) | 0.228 |  |  | |
| Previous pneumocystis pn. | 47 (8.9%) | 4 (7.7%) | 0.974 |  |  | |
| Previous atypical mycobac. pn. | 6 (1.1%) | 1 (1.9%) | 0.483 |  |  | |
| Previous tuberculosis pn. | 9 (1.7%) | 0 (0.0%) | 1 |  |  | |
