# Supplementary material for: COPD in HIV-Infected Patients: CD4 Cell Count Highly Correlated
Source: PLoS One. 2017 Jan 5;12(1):e0169359. doi: 10.1371/journal.pone.0169359 (PMC5215875; doi:10.1371/journal.pone.0169359)
Supplement: S2 Table — (DOCX) [file pone.0169359.s003.docx]

**S3 Table. Results of screening test for the 184 conventional pulmonary function testing performed.**

| Spirometry | Performed | Not performed |
| --- | --- | --- |
| Questionnaire positive | 153 | 428 |
| COPD-6 test positive | 66 | 515 |
| Questionnaire and COPD-6 test positive | 32 | 549 |
